# New Anthelmintic Remedy

**Published:** 1850-09

**Authors:** 


*New Anthelmintic Remedy.*—Dr. Budd, of Kings' College Hospital, L› don, Eng., has lately employed with success for the expulsion of tænia, a new remedy called *Kousso*, [Brayera Anthelmintica, of the natural order Rosaceæ.] A report favorable to the anthelmintic properties of this plant, was several years ago adopted by the Academy of Medicine of Paris, and, also, by the Academy of Sciences.

The parts of the plant used, are the flowers reduced to a fine powder, and given suspended in lukewarm water. The dose is not given in any of the accounts which have as yet fallen under notice. Several cases are reported in the Medical Times, in which it appeared to exert a prompt and efficient action in expelling tænia, after other remedies had been administered without effect.

